# The effect of mechanical canopy reduction on big sagebrush plant communities

**DOI:** 10.1002/eap.70056

**Published:** 2025-06-19

**Authors:** Phoebe L. Ferguson, Trace E. Martyn, Michelle C. Downey, James M. Fischer, Ingrid C. Burke, William K. Lauenroth

**Affiliations:** ^1^ School of the Environment Yale University New Haven Connecticut USA; ^2^ Eastern Oregon Agricultural Research Center – Union Experiment Station, Department of Animal and Rangeland Sciences Oregon State University Corvallis Oregon USA; ^3^ Forestry Manager Fort Garland Colorado USA

**Keywords:** arid and semiarid ecosystems, *Artemisia tridentata*, disturbance ecology, dryland vegetation, long‐term recovery, two‐layer hypothesis

## Abstract

A major conservation challenge in the western United States is implementing management treatments that reduce fire risk, control for invasive species, and maintain herbaceous understories in big sagebrush ecosystems. Studies have found that mechanical treatment of big sagebrush can reduce fire risk and promote herbaceous understories, but a consensus on the long‐term impacts of big sagebrush reduction remains unclear. We used a time series (20 years) of treated sites to understand the short‐ and long‐term response of herbaceous plants and shrubs to mastication treatment in big sagebrush plant communities of south‐central Colorado. We found that mastication to a height of 15 cm significantly reduced big sagebrush cover and increased perennial grass cover in the short term. The significant increase in perennial grass cover on recently treated (1–2 years) sites was largely attributed to C_3_ rather than C_4_ perennial bunchgrasses. Recently treated sites had greater annual plant cover and density than untreated sites. However, on sites treated more than 2 years ago, there was no significant difference between perennial grass and annual plant cover or density. Perennial forb cover and density was not affected by treatments. Initially reduced by nearly 80%, big sagebrush cover returned at a rapid and constant rate over time and returned to a statistically indistinguishable cover from the untreated sites within 8–10 years while height recovered slowly. Our results underscore the resilience of big sagebrush to partial canopy removal and emphasize the long‐term dynamics following treatment.

## INTRODUCTION

Big sagebrush ecosystems have undergone extensive habitat loss and degradation in the past century and are vulnerable to conversion to invasive annual grasslands, increased fire intensity, conifer expansion, and climate change (Davies, Boyd, et al., 2011; Knick & Connelly, 2011; Manier et al., 2013). An active area of research in big sagebrush plant communities asks how land treatments can be used to reduce woody fuels, mitigate invasive species expansion, and improve forage and wildlife habitat (Chambers et al., 2021; Copeland et al., 2018; Ellsworth et al., 2022). Mechanical canopy reduction of big sagebrush cover is one land treatment that may reduce fire risk, increase perennial grass and forb cover, and allow big sagebrush to recover over time. The recovery of big sagebrush post treatment is crucial, as less than half of the original expanse of big sagebrush ecosystems remains (Knick & Connelly, 2011; Manier et al., 2013; Pyke, 2011). To understand the trade‐offs between reducing woody fuels and preserving existing big sagebrush habitats, it is essential to evaluate the longevity and effectiveness of these treatments (Ellsworth et al., 2022). Despite their widespread implementation across the western United States, the long‐term effectiveness of land treatments in increasing forage and maintaining big sagebrush cover remains uncertain (Copeland et al., 2018; Munson et al., 2020; Pilliod et al., 2017).

Land treatments in the western United States are primarily used to reduce wildfire risk, enhance herbaceous cover and structural complexity, and improve habitat for wildlife (Manier et al., 2013; Munson et al., 2020; Pilliod et al., 2017). Compared to land treatments such as prescribed fire or chemical applications, mechanical treatments have a faster recovery time of big sagebrush cover, are easier to control, and reduce soil erosion (Davies et al., 2012a; Hess & Beck, 2012; Pyke et al., 2014). Mechanical treatments reduce fire rate of spread and reaction intensity, but their impacts on plant community composition depend on several factors, including site‐specific characteristics and the nature of the treatment applied (Chambers et al., 2021; Ellsworth et al., 2022; Riginos et al., 2019). Mechanical treatment type and height influence recovery of big sagebrush because they do not re‐sprout and must rely on perennating buds remaining after treatment in order to grow and recover (Bilbrough & Richards, 1993; Pyke et al., 2022). Some mechanical treatments (bulldozing, blading, chaining, cabling, railing, and pipe harrowing) uproot shrubs, while others (roller chopping, shredding/mulching, and mowing) reduce shrub canopies but do not uproot entire shrubs and leave canopies partially intact (Beck et al., 2012; Dahlgren et al., 2006; Vallentine, 1989). If treatments do not kill or uproot individuals, they may have minimal impact on the distribution of soil water resources over the long term if root systems remain intact. This should allow for rapid big sagebrush recovery after treatment with little effect on density (Pyke et al., 2014).

Mechanical treatments in big sagebrush ecosystems are typically applied as low to intermediate pulse disturbances that reduce big sagebrush cover in dense stands while leaving herbaceous understories intact and small shrubs in place (Chambers et al., 2021; Davies et al., 2009; Hess & Beck, 2012; Pechanec et al., 1965). Theoretically, mechanically reducing big sagebrush canopies can create a pulse of resources for competing species, increasing perennial grass and forb abundance, which can improve forage for wildlife and in some cases increase site resistance to annual grass invasion (Reisner et al., 2013; Riginos et al., 2019). Many obligate wildlife species, such as the greater sage‐grouse (*Centrocercus urophasianus*) and the Gunnison sage‐grouse (*Centrocercus minimus*), rely on big sagebrush habitat, which contains a diversity of native perennial grasses and forbs and abundant sagebrush cover (Crawford et al., 2004; Manier et al., 2013; Payton et al., 2011). However, most studies have found that big sagebrush canopy reduction increased herbaceous cover but only in the short term (Hess & Beck, 2012; Monaco & Gunnell, 2020; Pyke et al., 2022; Riginos et al., 2019; Swanson et al., 2016).

It is still unclear if treatments are effective at enhancing big sagebrush habitat for sagebrush‐dependent wildlife (Hess & Beck, 2014) or why increases in herbaceous cover are short‐lived. Perennial forb response has been especially variable; some studies have found little to no effect, while others have found that mechanical treatments decreased perennial forb abundance and increased annual forbs and grasses (Hess & Beck, 2014; Riginos et al., 2019). It is essential to determine the impact of mechanical treatments on various plant functional types, such as big sagebrush, cool‐season and warm‐season perennial grasses, perennial forbs, and annual plants. These functional types may exhibit different responses to treatments over time, which can offer valuable insights into the competitive interactions that sustain plant community composition over the long term.

Long‐term (>10 years) post‐treatment monitoring is needed to capture the full effects of management on plant community properties, particularly in dryland systems with long‐lived perennial species (Copeland et al., 2019). However, few mechanical treatment studies have monitored responses over a time period relevant to big sagebrush recovery (>10 years), particularly in the southwestern United States (Copeland et al., 2019; McIver et al., 2014). Of land treatments conducted on Bureau of Land Management lands between 1940 and 2010 in the southwestern United States, only 9.5% of projects included any post‐treatment monitoring (Copeland et al., 2018; Munson et al., 2020). Additionally, while treatment type plays a key role in plant community recovery, recent studies used harrow or roto‐cutter treatments that reduce sagebrush cover by approximately 50%. Fewer studies have examined the effects of mastication on big sagebrush plant communities. Mastication uses heavy machinery to chop and shred above‐ground plant material. It is commonly used to target trees in big sagebrush plant communities that are experiencing woodland expansion but can be used to reduce big sagebrush cover. The timing and amount of big sagebrush mortality from mastication influence the recovery trajectory of the plant community and can have long‐lasting effects on ecosystem services (Svejcar et al., 2014). It is therefore essential to determine the extent to which big sagebrush recovers after mastication and the effect of mastication on plant community composition and structure over time.

To address these gaps, we investigated how mastication influenced plant community dynamics in big sagebrush plant communities of south‐central Colorado. We addressed three questions: (1) What are the initial, short‐term effects of mastication on cover and density of big sagebrush, perennial grasses, and perennial forbs? (2) Does mastication change the long‐term density and cover of big sagebrush? (3) How do perennial grasses and forbs respond to mastication over time? To answer these questions, we characterized plant community composition and site conditions on paired treated and untreated sites in the San Luis Valley of Colorado.

## METHODS

### Site description and background information

The study area is at the edge of the Sangre De Cristo Mountains and the San Luis Valley in the semiarid region of south‐central Colorado (37°25′44″ N, 105°26′2″ W). In general, big sagebrush plant communities in south‐central Colorado receive summer precipitation from the North American monsoon and undergo variable wet and dry years from the El Niño‐Southern Oscillation (Loik et al., 2004). The 20‐year average annual precipitation was 338 mm (263–533 mm) and annual temperature was 6.2°C (5.2–6.6°C) between 2003 and 2022 (Appendix S1: Figure S1; PRISM Climate Group, 2004). Winter precipitation increases through April, then decreases in the dry season between May and June (Appendix S1: Figure S1). Precipitation then peaks from July to mid‐September due to the North American monsoon (Appendix S1: Figure S1; Adams & Comrie, 1997; Higgins et al., 1997). Nearly 60% of annual precipitation is received between April and September.

The plant communities in the study area are dominated by Wyoming big sagebrush (*Artemisia tridentata* Nutt. ssp. *wyomingensis* Beetle & Young) with an understory of mixed perennial grasses and forbs. Common perennial grasses include blue‐grama (*Bouteloua gracilis* (Willd. ex Kunth) Lag. ex Griffiths), squirreltail (*Elymus elymoides* (Raf.) Swezey), western wheatgrass (*Pascopyrum smithii* (Rydb.) Á. Löve), needle‐and‐thread (*Hesperostipa comata* (Trin. & Rupr.) Barkworth), and prairie Junegrass (*Koeleria macrantha* (Ledeb.) Schult.). Common annuals include prickly Russian thistle (*Salsola tragus* L.). Common other shrubs or subshrubs include yellow rabbitbrush (*Chrysothamnus viscidiflorus* (Hook.) Nutt.), broom snakeweed (*Gutierrezia sarothrae* (Pursh) Britton & Rusby), slender buckwheat (*Eriogonum microthecum* Nutt), and prairie sagewort (*Artemisia frigida* Willd.). Common forbs include pingue rubberweed (*Hymenoxys richardsonii* (Hook.) Cockerell) and shaggy fleabane (*Erigeron pumilus* Nutt.).

We used 10 × 10 m plots to sample 40 pairs of treated (i.e., masticated) and untreated sites in big sagebrush plant communities during June–August 2022. Sites were masticated between 2003 and 2022 using a Caterpillar 586C Site Prep Tractor with a BR624 Brushcutter attachment that removed shrub canopies to a height of approximately 15 cm. We selected sites based on treatment year and the presence of an adjacent untreated plot (~100–250 m). All sites were excluded from livestock grazing and received no restoration treatments. We used space‐for‐time substitution to compare plant community composition on treated sites over time (Pickett, 1989).

Space‐for‐time substitutions assume that spatial and temporal variation are equivalent and are used when long‐term studies and repeated measurements are not feasible (Pickett, 1989). We used a series of sites that were treated during different years (i.e., chronosequence) to investigate the patterns of plant community composition change over time since treatment (Pickett, 1989). Sites were located over an area of nearly 65 km^2^ and the elevation ranged from 2480 to 2802 m. Distance between sites ranged from approximately 0.25–19 km. Plant community composition and site conditions of untreated sites can be found in Appendix S1: Tables S1 and S2.

### Site and soil characterization

We recorded slope, aspect, and a GPS location of the point of origin at each 10 × 10 m plot we sampled. We used GPS coordinates to extract elevation, annual precipitation, and mean annual temperature for each plot (Appendix S1: Figure S2; PRISM Climate Group, 2004; U.S. Geological Survey, 2023). We collected three randomly located soil samples at three depths (0–10, 10–20, and 20–30 cm) from each plot and used these to estimate soil texture. Texture was estimated using a modified hydrometer method, and we used a 2‐mm sieve to separate gravel and estimated the percent of gravel by mass (Bouyoucos, 1951).

### Plant community composition

We estimated percent plant canopy and ground cover within each 10 × 10 m plot using a modified Daubenmire method (Daubenmire, 1959). We created a grid using distance (in centimeters) along the 0° and 90° azimuth of the plot and placed pin‐flags at 30 randomly generated and nonoverlapping coordinates. At each pin‐flag, we positioned a 20 × 50 cm quadrat so that it was oriented toward the north with the pin‐flag located in the upper right‐hand corner. We used modified Daubenmire cover classes (0 = 0, 1 = 0%–1%, 2 = 1%–2%, 3 = 2%–5%, 4 = 5%–10%, 5 = 10%–25%, 6 = 25%–50%, 7 = 50%–75%, 8 = 75%–95%, 9 = 95%–100%) to visually estimate plant species cover and ground cover in each quadrat. We estimated cover for seven surface categories: bare ground, moss, woody litter (any material from woody plants), herbaceous litter, vagrant lichen, biotic crust, and rock (>5 mm). In addition to percent cover, we estimated average height and counted the number of individuals of each plant species that had >50% cover and were rooted within each quadrat. To determine the average height and density for each species at the plot level, we calculated the sum of the heights and density counts and then divided by the total number of quadrats. For rhizomatous grasses, we counted each stem as one individual. We used functional traits and USDA PLANTS database classifications to assign species to one of nine functional types: big sagebrush, other shrub, subshrub, tree, C_3_ perennial bunchgrass, C_4_ perennial bunchgrass, C_3_ rhizomatous grass, annual (annual grass and forb), and perennial forb (USDA & NRCS, 2022). A small number of sedges were found on some sites and were included as C_3_ perennial grasses. We used a separate category (standing dead) to estimate the percent cover of all rooted and nonliving plant material in each quadrat. To calculate mean percent cover for the ground surface and plant functional types at the plot level, we summed cover class midpoints in each group and divided by the total number of quadrats.

### Shrub density

We assigned size classes to each shrub in a 5 × 10 m subset of each plot. Size classes were based on the estimated diameter of a sphere equal to the volume of each plant (Renne et al., 2019). For shrub stumps in masticated sites that were 0% living and <5 cm in height, we recorded a size class of zero. For each individual shrub, we estimated the percent of the canopy that was living in 10% increments (Renne et al., 2019). To avoid bias toward small individuals, we did not count shrubs in the 5 × 10 m subplot when their average canopy length, width, and height were <5 cm. We estimated big sagebrush recruitment by counting the number of big sagebrush seedlings (<5 cm) in a 1 × 10‐m belt transect across the center of the plot.

### Statistical analyses

We used one‐way ANOVA and Dunnett's comparisons to estimate how percent cover, density, or height of plant functional type or soil surface cover differed (α = 0.05) between treated groups and one untreated group using the *stats* and *multcomp* package in R version 4.3.2 (Hothorn et al., 2008; R Core Team, 2023). We used log or square root transformations of our response variables to improve normality (Appendix S1: Table S5). When assumptions of equal variance were not met, we used a Kruskal–Wallis test and Wilcoxon signed‐rank test for pairwise comparisons in the *stats* package in R (R Core Team, 2023). We grouped sites by years‐since‐treatment (1–2 years [*n* = 4], 3–4 years [*n* = 4], 8–10 years [*n* = 4], 11–15 years [*n* = 4], and 17–19 years [*n* = 4]) and compared response variables between treated groups and one untreated group (*n* = 20). If a site was treated during the winter or spring of the sampling year, we recorded the years‐since‐treatment as 1 year. To estimate the rate of recovery on treated sites, we used linear regression to estimate how big sagebrush percent cover and height changed over time (α = 0.05).

## RESULTS

### Short‐term effects of mastication on plant community composition

**FIGURE 1:**
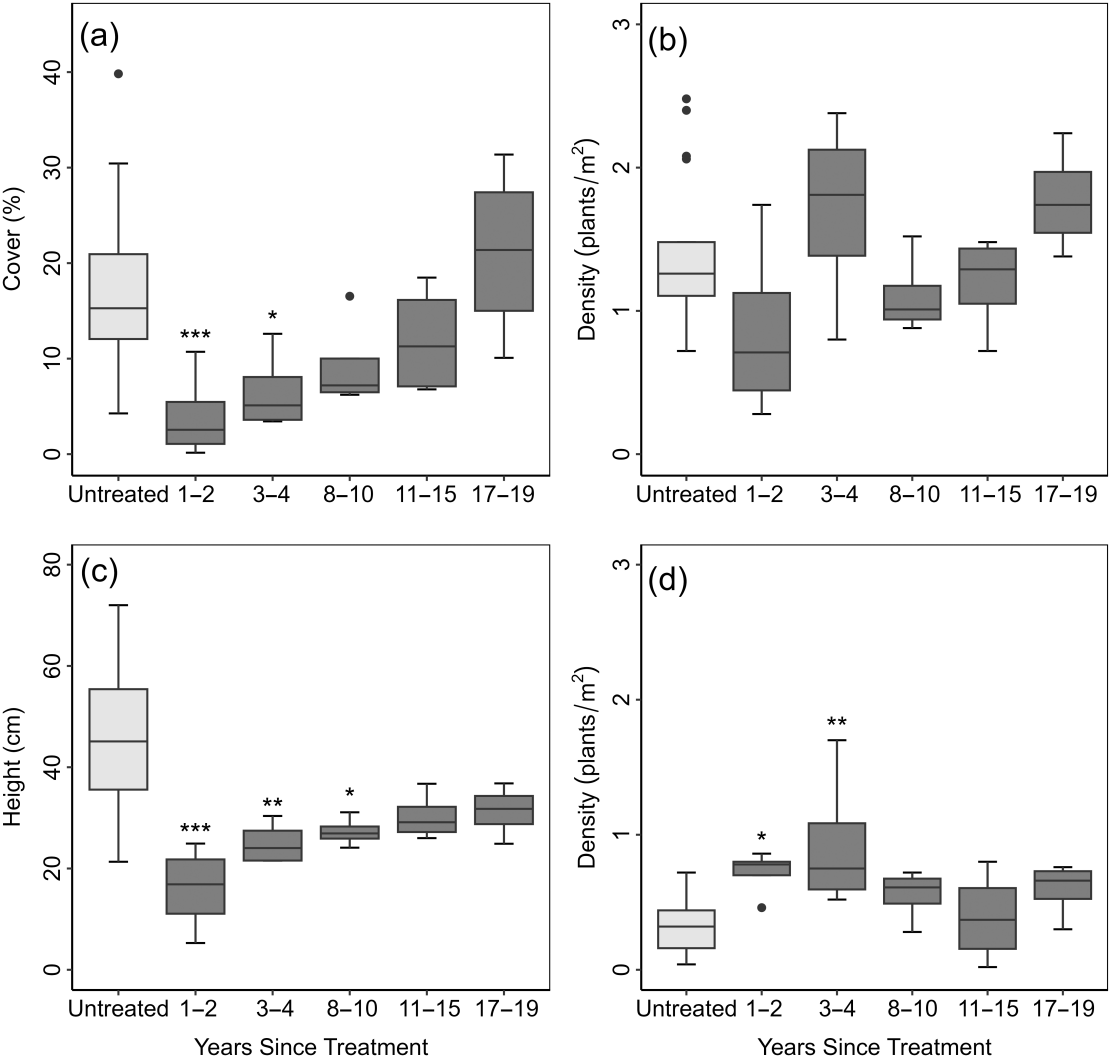
Big sagebrush cover (a), live density (b), average height (c), and dead density (d) on treated (dark gray) and untreated sites (light gray). Solid lines are medians. Whiskers show the spread of data and boxes represent the interquartile range. Outliers are represented by solid dots. Statistically significant differences between untreated (*n* = 20) and treated sites (1–2 [*n* = 4], 3–4 [*n* = 4], 8–10 [*n* = 4], 11–15 [*n* = 4] or 17–19 [*n* = 4] years since treatment) are represented by asterisks (*p < 0.05; **p < 0.01; ***p < 0.001).

**FIGURE 2:**
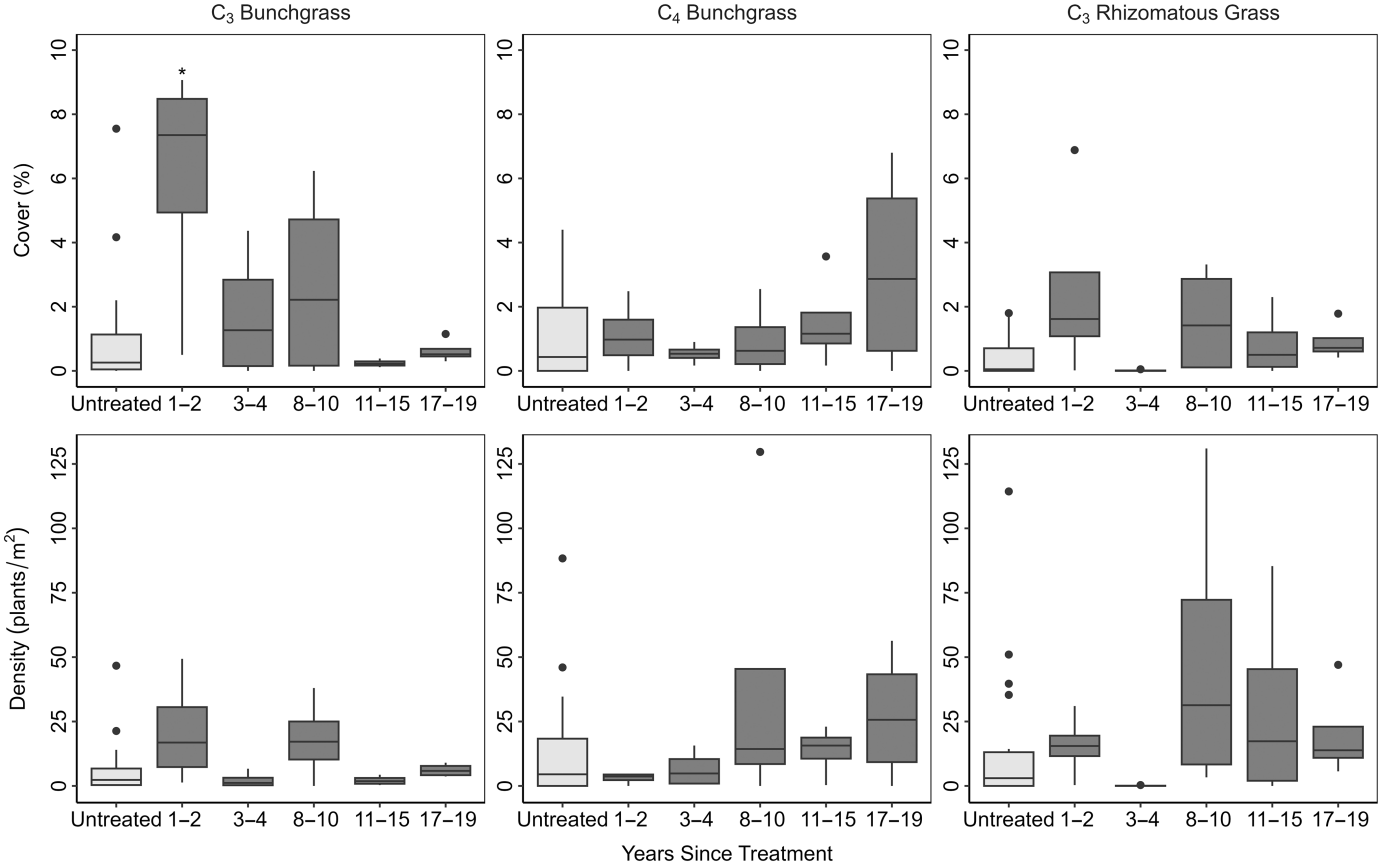
C_3_ bunchgrass, C_4_ bunchgrass, and C_3_ rhizomatous grass cover and density on treated (dark gray) and untreated sites (light gray). Solid lines are medians. Whiskers show the spread of data and boxes represent the interquartile range. Outliers are represented by solid dots. Statistically significant differences between untreated (*n* = 20) and treated sites (1–2 [*n* = 4], 3–4 [*n* = 4], 8–10 [*n* = 4], 11–15 [*n* = 4] or 17–19 [*n* = 4] years since treatment) are represented by asterisks (*p < 0.05; **p < 0.01; ***p < 0.001).

**FIGURE 3:**
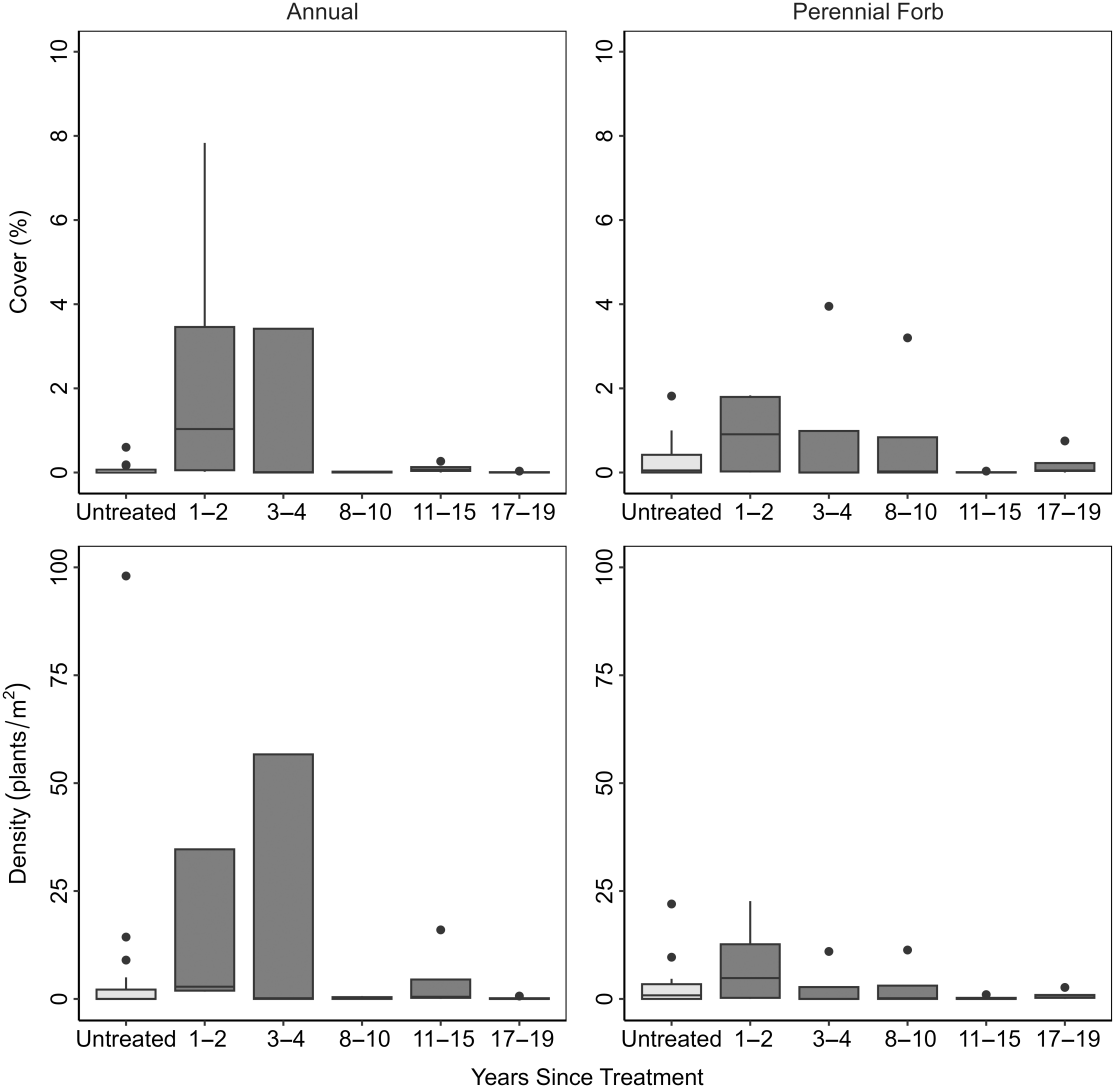
Annual forb and grass and perennial forb cover and density on treated (dark gray) and untreated sites (light gray). Solid lines are medians. Whiskers show the spread of data and boxes represent the interquartile range. Outliers are represented by solid dots. Statistically significant differences between untreated (*n* = 20) and treated sites (1–2 [*n* = 4], 3–4 [*n* = 4], 8–10 [*n* = 4], 11–15 [*n* = 4] or 17–19 [*n* = 4] years since treatment) are represented by asterisks (*p < 0.05; **p < 0.01; ***p < 0.001).

**FIGURE 4:**
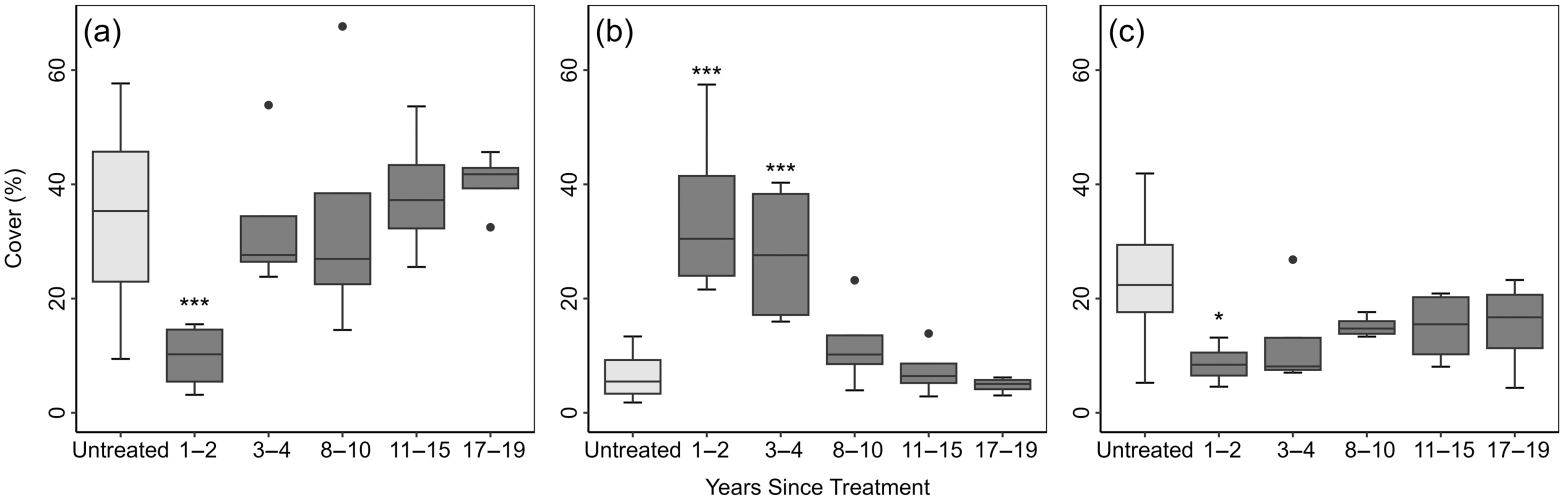
Bare ground (a), woody litter (b), and herbaceous litter (c) cover on treated (dark gray) and untreated sites (light gray). Solid lines are medians. Whiskers show the spread of data and boxes represent the interquartile range. Outliers are represented by solid dots. Statistically significant differences between untreated (*n* = 20) and treated sites (1–2 [*n* = 4], 3–4 [*n* = 4], 8–10 [*n* = 4], 11–15 [*n* = 4] or 17–19 [*n* = 4] years since treatment) are represented by asterisks (*p < 0.05; **p < 0.01; ***p < 0.001).

Mastication significantly reduced big sagebrush cover from 17% to 4% in the first 1–2 years post treatment (Figure 1, Appendix S1: Table S3). Live big sagebrush density did not significantly differ from untreated sites, although it was slightly lowered (1.4–0.9 plants/m^2^) (Figure 1, Appendix S1: Table S4). Total perennial grass cover significantly increased from 3% to 10% (Figure 2, Appendix S1: Table S3). Percent cover of annuals (grasses and forbs) increased from 1% to 3% and density increased from 3 to 34 plants/m^2^ but did not significantly vary from untreated sites (Figure 3, Appendix S1: Table S3). Perennial forb and other shrub cover and density did not significantly vary between treated and untreated groups 1–2 years after treatment (Figure 3, Appendix S1: Table S5). Mastication significantly increased woody litter (7%–35%) and decreased herbaceous litter (23%–9%) (Figure 4, Appendix S1: Table S3). Bare ground significantly decreased from the average of untreated sites (35%–10%) (Figure 4, Appendix S1: Table S5).

### Recovery of big sagebrush over time

**FIGURE 5:**
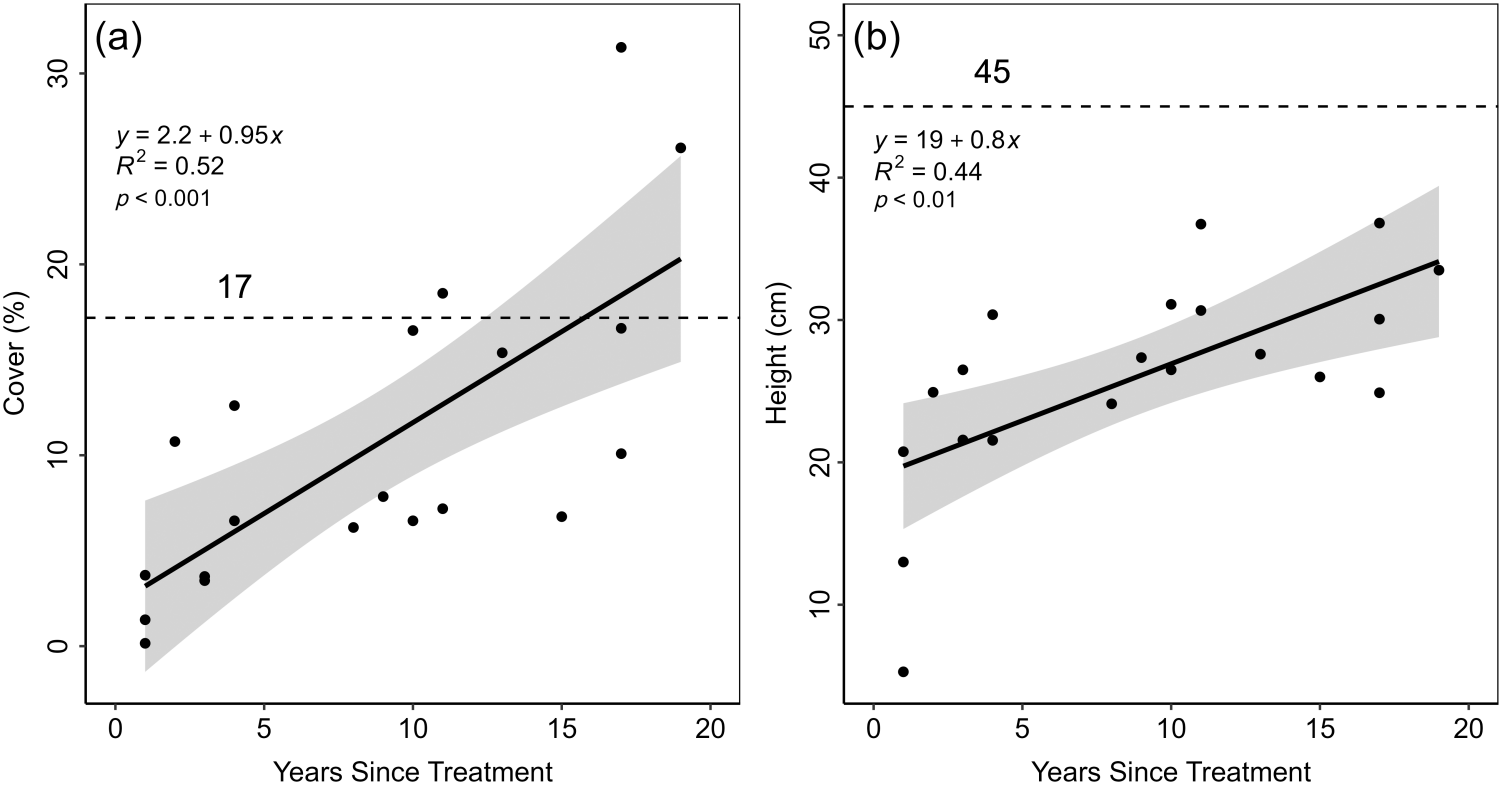
Big sagebrush percent cover (a) and height (b) on treated sites over time (*n* = 20) and 95% confidence bands (light gray). The average percent cover and height on untreated sites are represented by dashed lines (*n* = 20).

Big sagebrush cover on masticated sites was significantly reduced but returned to levels comparable to untreated sites (Figures 1 and 5). After treatment, cover increased, and after 3–4 years, big sagebrush cover was not statistically significantly different from untreated sites (Figure 1, Appendix S1: Table S5). Although average big sagebrush height gradually increased after mastication, it remained significantly lower than untreated sites until 11–15 years after treatment (Figure 1, Appendix S1: Table S5). Mastication significantly reduced both cover and height in the short term, but big sagebrush live density did not significantly differ from untreated sites (Figure 1, Appendix S1: Table S5). While the density of 100% dead big sagebrush increased shortly after treatment, it did not significantly differ from untreated sites after 3–4 years (Figure 1, Appendix S1: Table S5). After treatment, big sagebrush cover increased at an approximate rate of 1% per year, and height increased at a rate of about 1 cm per year (Figure 5). This suggests that if recovery is continuous, it would take 15–16 years for big sagebrush cover to return to the average of the untreated sites and close to 30 years for height to return to the average of untreated sites (Figure 5).

### Plant community response to treatment over time

C_3_ perennial bunchgrass cover 1–2 years after treatment was significantly higher than the average of all untreated sites but returned to levels comparable to untreated sites within 3–4 years (Figure 2). C_4_ perennial bunchgrass and rhizomatous grass cover and density did not significantly differ between treated and untreated sites (Figure 2, Appendix S1: Table S5). Although annual cover and density increased shortly after treatment, they did not significantly differ from untreated sites over time (Figure 3, Appendix S1: Table S5). We found no significant response in perennial forb cover or density over time (Figure 3, Appendix S1: Table S5). Woody litter remained significantly higher than untreated sites until 8‐10 years after treatment (Figure 4, Appendix S1: Table S5). In the short term, herbaceous litter and bare ground decreased but returned to comparable pre‐treatment levels after 2 years (Figure 4, Appendix S1: Table S5).

## DISCUSSION

We aimed to understand the short‐ and long‐term response of herbaceous plants and shrubs to mastication treatment in big sagebrush plant communities in south‐central Colorado. We found that mastication increased perennial grass cover in the short term and that big sagebrush cover rapidly returned to pre‐treatment levels. C_3_ grasses were more responsive to treatments than C_4_ grasses. Big sagebrush cover returned at a rapid and constant rate, whereas height recovery was slower and delayed over time. Big sagebrush and herbaceous species density were not affected by treatment, and perennial grass cover decreased after 2 years as big sagebrush cover returned.

### Short‐term effects of mastication on plant community composition

Mastication reduced big sagebrush cover by nearly 80% in our study but had no significant effect on live density. We estimated that mastication killed approximately 35% of big sagebrush plants, but the treatment left smaller plants in place and most individuals that had leaves and stems left intact were able to quickly recover. The positive response in perennial grass cover shortly after treatment suggests a competitive release from big sagebrush reduction (Adler et al., 2018). This is unsurprising as perennial grasses are strong competitors for soil resources in the upper 0–30 cm and may rapidly respond to changes in resource availability after treatment (Sala et al., 1997). Our results were similar to Davies et al. (2012b), which found that mechanical removal in dense Mountain big sagebrush sites increased herbaceous cover 1.7‐fold and 1.5‐fold 2 and 3 years after mechanical treatment, while perennial forbs and annual grass did not respond.

Big sagebrush mastication did not affect perennial forb cover and density (Davies et al., 2012b; Hess & Beck, 2014). Prevéy et al. (2010) suggested that big sagebrush may have a net positive effect on native forb growth, which may explain why mastication did not increase perennial forb cover. The short‐term increase in annuals was likely because these species are adept at responding quickly and capitalizing on increased available resources after disturbance. Our results support the results of Davies, Bates et al. (2011) and demonstrate that careful attention to the presence of annual species pre‐treatment is important for understanding the potential response of annuals post mastication. For example, the quantity of annual grass cover before treatment is important for determining how vulnerable treated sites may be to annual grass invasion. *Bromus tectorum* was found on three sites across our study area and only one site in the 1‐ to 2‐year treatment group. Davies, Bates et al. (2011) found that mechanical treatments slightly increased exotic annuals in Wyoming big sagebrush plant communities with intact understory vegetation in Oregon. They cautioned that mechanical treatments could still increase the risk of invasion (Davies, Bates, et al., 2011). It is unclear in our study how increased cover in annual species may have influenced perennial grass cover. However, when perennial vegetation recovers rapidly after disturbance, the likelihood of invasion is generally reduced (Davies et al., 2012b).

### Recovery of big sagebrush over time

Big sagebrush cover returned rapidly after treatment while height recovered slowly. Big sagebrush cover rebounded within 8–10 years after treatment. Our results were similar to other studies that found little effect of similar mechanical treatments on big sagebrush cover or density after 3 years (Chambers et al., 2021; Pyke et al., 2014, 2022). When reduced by nearly 50%, Rau et al. (2014) found that big sagebrush cover returned 3 years post treatment. Our sites were reduced by nearly 80%, but big sagebrush cover was not significantly different from untreated sites within 8–10 years. However, it was still lower than the average of untreated sites (Figure 1, Appendix S1: Table S3). It may take closer to 15–16 years to fully recover to the average of pre‐treatment levels in big sagebrush plant communities of south‐central Colorado (Figure 3). Overall, even after significant disturbance and increased perennial grass cover, big sagebrush cover rapidly returned.

In plant communities where woody and herbaceous species interact, the dominance or coexistence of plant functional types is influenced by the spatial and temporal distribution of available water in the soil profile (Sala et al., 1997). Although our sites were treated to a height of approximately 15 cm, big sagebrush that retained portions of its canopy after treatment, or small plants that were left relatively intact, likely retained better access to deeper soil water resources than other competing plant functional types. Perennial grasses have roots that are concentrated in the upper 30 cm of soil, while woody plants have taproots that can access water deeper in the soil profile (Sala et al., 1997; Schlaepfer et al., 2012). This partitioning of roots in the soil profile allows these functional groups to coexist through differentiation of water acquisition. Inouye (2006) found that soil moisture was higher on shrub‐removal plots dominated by perennial grasses, particularly at depths of 80–180 cm. This suggests that perennial grasses did not extract as much moisture as shrubs, especially in deeper soil layers (Inouye, 2006). Further investigating the effect of mechanical treatments on soil water may help explain plant community response to mastication and treatment longevity.

### Plant community response over time

Total perennial grass cover initially increased in response to mastication but decreased as big sagebrush cover returned. We found that C_3_, C_4_, and rhizomatous grass cover and density responded differently to mastication over time since treatment. Treatments had a greater effect on C_3_ rather than C_4_ perennial bunchgrasses. *B. gracilis* is the common C_4_ species on our sites, and it is a relatively drought‐tolerant species. Although established *B. gracilis* individuals have been shown to rapidly respond to pulses of resources (Sala & Lauenroth, 1982), its ability to spread vegetatively or by seed is slow and may explain why C_4_ bunchgrass cover was less impacted than C_3_ bunchgrass cover in the short term.

C_3_ bunchgrass cover had a greater initial response to mastication, but the effect decreased over time. The short‐lived increase in perennial grass cover is likely related to the differentiation of available soil water mentioned above. Big sagebrush typically relies on soil water recharge from cool‐season precipitation (Schlaepfer et al., 2012) and after mastication treatment, cool‐season grasses may be more likely than warm‐season grasses to capitalize on increased soil water availability in the spring. For example, in a shrub‐removal experiment, Prevéy et al. (2010) found that in the first year after treatment, shallow‐soil water content (20 cm) was greater on treated sites in the late spring and early summer. However, the effect diminished as the summer progressed and was not significantly different in the following year.

We found short‐term increases in annual species, but cover and density of annual forbs and grasses did not significantly differ from untreated sites over time. Summers and Roundy (2018) found that in mechanically treated Wyoming big sagebrush plant communities with initially low cheatgrass cover, cheatgrass increased to a maximum of 1.9% on one‐way harrow treatments. Other studies have shown little increase in annual grass from mechanical treatments (Riginos et al., 2019; Swanson et al., 2016). Similar to other studies, we found that mastication had no effect on perennial forb cover or density over time (Hess & Beck, 2014; Riginos et al., 2019).

Mastication increased woody litter and decreased bare ground, but soil surface cover returned to pre‐treatment levels within 10 years. While mastication altered vegetation cover and ground cover, it is unclear how changes in these microsite conditions may have influenced plant cover and density in our study. It is likely that mastication altered fuel dynamics by redistributing above‐ground woody material to the soil surface and reducing big sagebrush cover and height (Chambers et al., 2024; Ellsworth et al., 2022). Ellsworth et al. (2022) found that mechanical treatments reduced modeled flame length 10 years after treatment. Mastication treatments likely reduced wildfire reaction intensity and created a mosaic of recovering big sagebrush across the landscape, but they had limited impact on perennial forage over the long term.

## LIMITATIONS

Our space‐for‐time substitution study design limits our ability to understand variation in treatment responses between sites. Mastication significantly increased perennial grass cover on recently treated sites, but the sites had higher elevation, higher C_3_ perennial grass density, and lower C_4_ perennial bunchgrass density than many of our other untreated sites (Appendix S1: Table S2). This variation in pre‐existing site conditions among sites could play a crucial role in explaining treatment outcomes (Chambers et al., 2014; Riginos et al., 2019). The effectiveness of treatments in enhancing perennial forage might be affected by the initial abundance of plants that compete most successfully with big sagebrush, especially during times of high soil water availability in the spring. Additionally, our study design also limits our understanding of how weather before and after treatment affects plant community response to treatments over time. To further understand how mastication influences plant community composition over the long term, it will be important to understand how climate change will influence the recovery trajectory of big sagebrush plant communities.

## CONCLUSIONS

Our results indicated that big sagebrush is resilient to partial canopy removal by mastication and that treatments increased perennial grasses in the short term. Although mastication significantly reduced cover of the dominant species, big sagebrush, plant community composition returned to pre‐treatment levels after 10 years. Big sagebrush rapidly recovered after treatment, likely due to niche separation between potentially competing plant functional types; studying the effect of mastication on the availability of water in the soil profile may clarify the mechanisms driving the plant community response to treatment. We did not find significant increases in annual grass or forb cover over the long term, but caution should be taken based on pre‐existing site conditions as it is unclear how annual species will respond across sites or regions. While our study was limited to one region, it provides important insight into treatment effectiveness in an area under‐represented in the literature.

## AUTHOR CONTRIBUTIONS

William K. Lauenroth, Michelle C. Downey, and James M. Fischer conceived the study. Phoebe L. Ferguson and Trace E. Martyn implemented the study. Phoebe L. Ferguson collected the data, did the analyses, and wrote the first draft. All authors participated in completing the final draft.

## CONFLICT OF INTEREST STATEMENT

The authors declare no conflicts of interest.

## Supporting information


Appendix S1.

## Data Availability

Data (Ferguson, 2025) are available in Figshare at https://doi.org/10.6084/m9.figshare.26046418.v1.
