# Supplementary material for: The effect of mechanical canopy reduction on big sagebrush plant communities
Source: Ecol Appl. 2025 Jun 19;35(4):e70056. doi: 10.1002/eap.70056 (PMC12178150; doi:10.1002/eap.70056)
Supplement: Supplementary file 1 — Appendix S1. [file EAP-35-e70056-s001.pdf]

Supporting Information

Manuscript: The effect of mechanical canopy reduction on big sagebrush plant communities

Authors: Phoebe L. Ferguson, Trace E. Martyn, Michelle C. Downey, James M. Fischer, Ingrid C. Burke, William K. Lauenroth

Journal Name: Ecological Applications

Figure S1: Walter and Leith climate diagram (a) (Walter and Leith, 1967; Guijarro, 2016) and average annual precipitation across sites between 2003 and 2022 (b) (PRISM Climate Group, 2004) 20-year average annual precipitation (338 mm) shown as a dashed line.

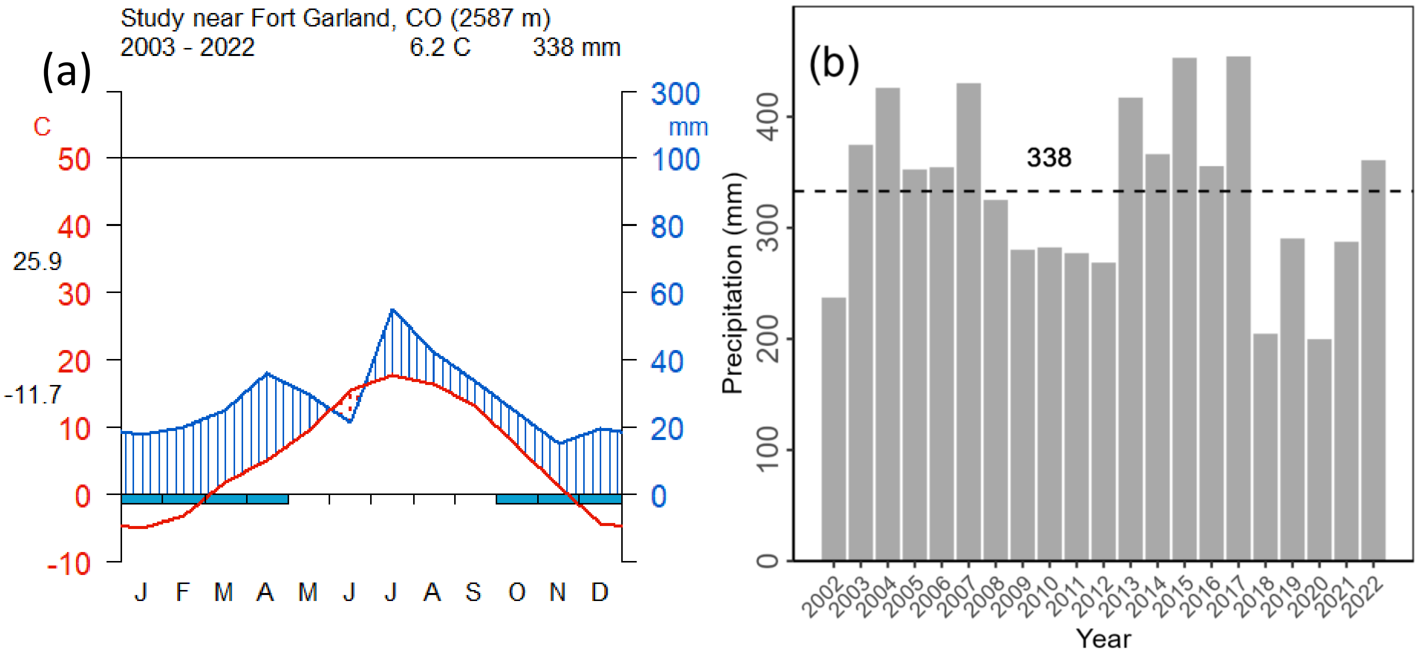

Figure S2: Elevation (a), percent sand (b), annual precipitation (c), and temperature (d) on untreated (light grey, n = 20) and treated sites (dark grey, n = 20) between 2003 - 2022. Solid lines are medians and dashed lines are means. Whiskers show the spread of data and boxes represent the interquartile range. Outliers are represented by solid dots.

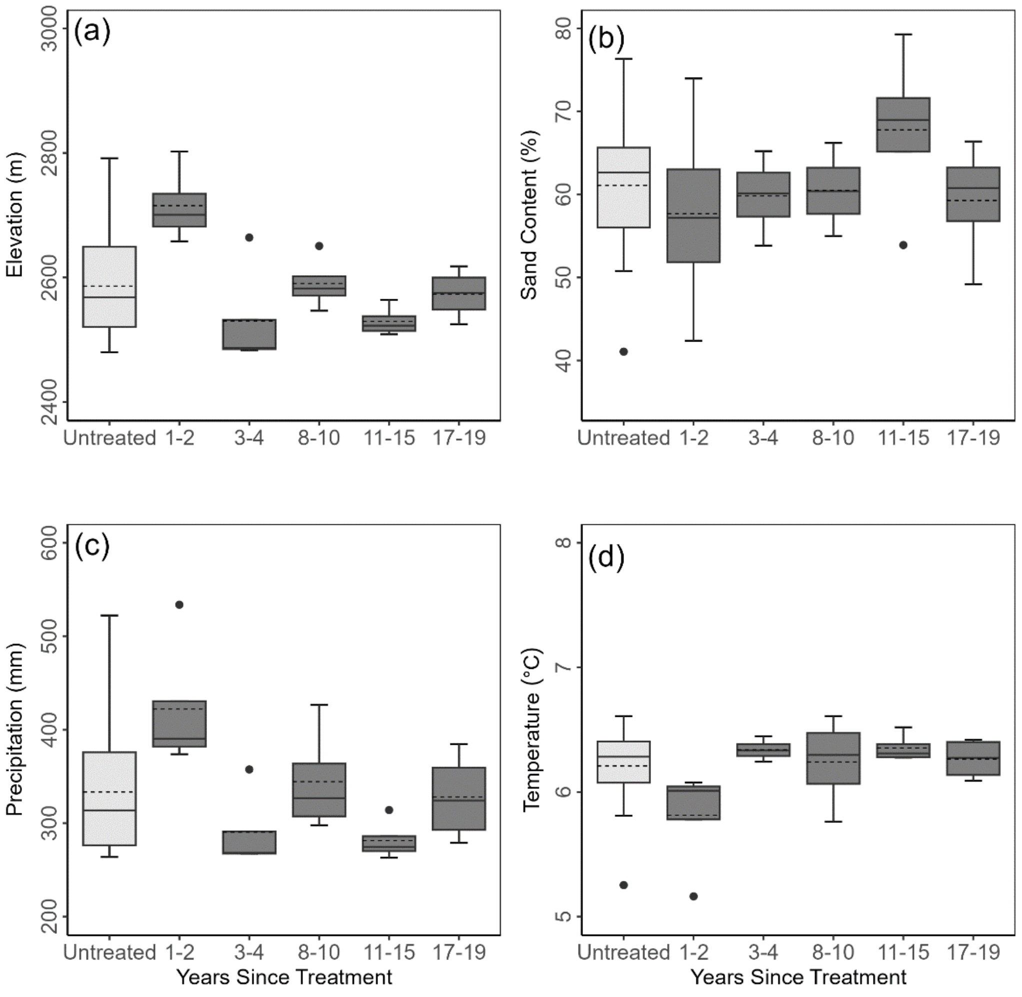

Table S1. Mean and standard error of cover of plant functional types and soil surface cover on untreated sites, grouped by their paired sites (1-2 (*n*=4), 3-4 (*n*=4), 8-10 (*n*=4), 11-15 (*n*=4), and 17-19 (*n*=4) years-since-treatment).

	Cover										
**Years**	**SAGE**	**PG**	**C3**	**RH**	**C4**	**PF**	**AN**	**SH**	**BG**	**WL**	**HL**
1-2	20.34 ± 2.95	2.63 ± 1.66	2.19 ± 1.79	0.24 ± 0.17	0.20 ± 0.17	0.48 ± 0.45	0.21 ± 0.13	0.32 ± 0.20	15.47 ± 2.32	7.48 ± 2.14	28.18 ± 5.33
3-4	14.73 ± 1.86	1.07 ± 1.01	0.28 ± 0.22	0.10 ± 0.10	0.69 ± 0.69	0.20 ± 0.20	0.02 ± 0.02	1.57 ± 0.71	47.46 ± 4.63	7.70 ± 2.09	22.67 ± 1.98
8-10	19.31 ± 7.60	2.98 ± 0.72	0.76 ± 0.50	0.55 ± 0.39	1.68 ± 0.97	0.48 ± 0.22	0.00 ± 0.00	1.12 ± 0.32	36.64 ± 8.08	4.98 ± 1.09	18.07 ± 6.52
11-15	15.17 ± 5.16	1.49 ± 0.89	0.06 ± 0.03	0.43 ± 0.43	1.00 ± 0.89	0.05 ± 0.04	3.02 ± 3.02	0.00 ± 0.00	37.94 ± 5.35	4.77 ± 1.86	19.36 ± 4.64
17-19	16.40 ± 2.51	5.00 ± 0.88	2.06 ± 0.77	0.83 ± 0.37	2.11 ± 0.86	0.27 ± 0.14	0.05 ± 0.04	2.07 ± 0.62	34.50 ± 4.70	7.41 ± 1.96	24.69 ± 6.17

SAGE big sagebrush (%), PG total perennial grass (%),C3 C_3 perennial bunchgrass (%), RH rhizomatous perennial grass (%), C4 C_4 perennial bunchgrass (%), PF perennial forb (%), AN annual (%), SH other shrub (%), BG bare ground (%), WL woody litter (%), HL herbaceous litter (%)

Table S2. Mean and standard error of plant functional type density on untreated sites, grouped by their paired sites (1-2 (*n*=4), 3-4 (*n*=4), 8-10 (*n*=4), 11-15 (*n*=4), and 17-19 (*n*=4) years-since-treatment).

				Density				
**Years**	**SAGE**	**SAGE_dead**	**C3**	**RH**	**C4**	**PF**	**AN**	**SH**
1-2	1.21 ± 0.13	0.24 ± 0.10	14.08 ± 10.93	5.58 ± 3.47	1.50 ± 1.29	5.83 ± 5.40	3.08 ± 2.02	0.30 ± 0.22
3-4	1.43 ± 0.24	0.36 ± 0.17	1.67 ± 1.35	2.92 ± 2.92	8.33 ± 8.33	1.17 ± 1.17	3.75 ± 3.53	0.49 ± 0.21
8-10	1.23 ± 0.04	0.27 ± 0.03	5.32 ± 3.04	15.92 ± 11.79	28.75 ± 20.16	3.50 ± 2.13	0.17 ± 0.17	0.52 ± 0.20
11-15	1.92 ± 0.41	0.47 ± 0.14	0.75 ± 0.53	28.67 ± 28.56	13.58 ± 10.98	1.00 ± 0.89	24.50 ± 24.50	0.14 ± 0.06
17-19	1.14 ± 0.11	0.28 ± 0.04	11.42 ± 3.97	21.92 ± 9.40	17.33 ± 7.00	2.25 ± 0.90	1.92 ± 1.20	0.54 ± 0.24

SAGE big sagebrush (m^2), SAGE_dead dead big sagebrush (m^2), C3 C_3 perennial bunchgrass (m^2), RH rhizomatous perennial grass (m^2), C4 C_4 perennial bunchgrass (m^2), PF perennial forb (m^2), AN annual (m^2), SH other shrub (m^2)

Table S3. Mean and standard error of cover of plant functional types and soil surface cover on treated (1-2 (*n*=4), 3-4 (*n*=4), 8-10 (*n*=4), 11-15 (*n*=4), and 17-19 (*n*=4) years-since-treatment) and untreated sites (*n*=20).

	Cover										
**Years**	**SAGE**	**PG**	**C3**	**RH**	**C4**	**PF**	**AN**	**SH**	**BG**	**WL**	**HL**
Untreated	17.19 ± 1.87	2.63 ± 0.53	1.07 ± 0.41	0.43 ± 0.14	1.13 ± 0.35	0.30 ± 0.11	0.66 ± 0.60	1.01 ± 0.25	34.40 ± 3.20	6.47 ± 0.80	22.59 ± 2.22
1-2	3.99 ± 2.36	9.71 ± 2.65	6.07 ± 1.94	2.53 ± 1.50	1.11 ± 0.53	0.91 ± 0.52	2.48 ± 1.84	0.75 ± 0.56	9.78 ± 3.02	35.00 ± 8.11	8.64 ± 1.83
3-4	6.56 ± 2.14	2.27 ± 1.09	1.72 ± 1.03	0.01 ± 0.01	0.53 ± 0.15	0.99 ± 0.99	3.41 ± 3.40	4.80 ± 3.94	33.23 ± 6.94	27.86 ± 6.45	12.52 ± 4.78
8-10	9.29 ± 2.44	5.17 ± 2.52	2.67 ± 1.53	1.56 ± 0.85	0.95 ± 0.57	0.81 ± 0.80	0.01 ± 0.01	0.03 ± 0.02	34.01 ± 11.61	11.88 ± 4.05	15.12 ± 0.95
11-15	11.96 ± 2.94	2.58 ± 0.77	0.24 ± 0.06	0.82 ± 0.52	1.51 ± 0.72	0.01 ± 0.01	0.10 ± 0.06	0.45 ± 0.45	38.42 ± 5.88	7.40 ± 2.32	14.98 ± 3.22
17-19	21.05 ± 4.76	4.66 ± 1.89	0.62 ± 0.18	0.91 ± 0.30	3.13 ± 1.62	0.21 ± 0.18	0.01 ± 0.01	0.13 ± 0.12	40.41 ± 2.80	4.83 ± 0.69	15.26 ± 4.14

SAGE big sagebrush (%), PG total perennial grass (%),C3 C_3 perennial bunchgrass (%), RH rhizomatous perennial grass (%), C4 C_4 perennial bunchgrass (%), PF perennial forb (%), AN annual (%), SH other shrub (%), BG bare ground (%), WL woody litter (%), HL herbaceous litter

Table S4. Mean and standard error of density of plant functional types on treated (1-2 (*n*=4), 3-4 (*n*=4), 8-10 (*n*=4), 11-15 (*n*=4), and 17-19 (*n*=4) years-since-treatment) and untreated sites (*n*=20).

	Density							
**Years**	**SAGE**	**SAGE_dead**	**C3**	**RH**	**C4**	**PF**	**AN**	**SH**
Untreated	1.38 ± 0.11	0.33 ± 0.05	6.65 ± 2.47	15.00 ± 6.21	13.90 ± 4.98	2.75 ± 1.15	6.68 ± 4.88	0.40 ± 0.09
1-2	0.86 ± 0.32	0.72 ± 0.09	21.08 ± 10.55	15.58 ± 6.26	3.00 ± 1.06	8.08 ± 5.32	33.75 ± 31.31	0.19 ± 0.10
3-4	1.70 ± 0.34	0.93 ± 0.27	2.25 ± 1.54	0.08 ± 0.08	6.50 ± 3.57	2.75 ± 2.75	56.50 ± 56.39	0.87 ± 0.67
8-10	1.10 ± 0.14	0.56 ± 0.10	18.08 ± 7.90	49.25 ± 29.36	39.58 ± 30.24	2.92 ± 2.81	0.25 ± 0.16	0.69 ± 0.33
11-15	1.19 ± 0.17	0.39 ± 0.17	2.08 ± 0.90	30.00 ± 19.82	13.67 ± 4.82	0.25 ± 0.25	4.25 ± 3.92	0.22 ± 0.15
17-19	1.77 ± 0.19	0.59 ± 0.10	6.08 ± 1.26	20.08 ± 9.19	26.92 ± 12.74	0.83 ± 0.62	0.17 ± 0.17	0.40 ± 0.17

SAGE big sagebrush (m^2), SAGE_dead dead big sagebrush (m^2), C3 C_3 perennial bunchgrass (m^2), RH rhizomatous perennial grass (m^2), C4 C_4 perennial bunchgrass (m^2), PF perennial forb (m^2), AN annual (m^2), SH other shrub (m^2)

Table S5. Analysis of variance testing for significant differences in response variables between untreated (*n*=20) and treated groups (1-2 (*n*=4), 3-4 (*n*=4), 8-10 (*n*=4), 11-15 (*n*=4), and 17-19 (*n*=4) years-since-treatment). Asterisks indicate significance (* p < 0.05, ** p < 0.01, *** p < 0.001).

**Response**	**Test**	***p*-value**	**X²**	**F-statistic**	**df**	**Post-hoc Test**	**1-2**	**3-4**	**8-10**	**11-15**	**17-19**
sqrt(SAGE)	ANOVA	<0.001 ***		5.7	5, 34	Dunnett's	<0.001 ***	0.022 *	0.300	0.784	0.953
SAGE_height_cm	ANOVA	<0.001 ***		7.9	5,34	Dunnett's	<0.001 ***	0.006 **	0.018 *	0.065	0.099
log(SAGE_live_density_m2)	ANOVA	0.571		2.4	5,34						
sqrt(SAGE_dead_density)	ANOVA	0.003 **		4.5	5,34	Dunnett's	0.042 *	0.002 **	0.407	0.996	0.259
sqrt(PG)	ANOVA	0.029 *		2.9	5,34	Dunnett's	0.006 **	1.000	0.684	1.000	0.726
log(C3)	ANOVA	0.023 *		3.0	5,34	Dunnett's	0.008 **	0.949	0.676	0.923	1.000
log(BG)	ANOVA	<0.001 ***		5.7	5,34	Dunnett's	<0.001 ***	1.000	1.000	0.967	0.863
log(WL)	ANOVA	<0.001 ***		11.6	5,34	Dunnett's	<0.001 ***	<0.001 ***	0.292	0.994	0.984
log(HL)	ANOVA	0.042 *		2.6	5,34	Dunnett's	0.020 *	0.164	0.834	0.673	0.527
log(C3_BU_density_m2)	ANOVA	0.137		1.8	5,34						
log(C4_density_m2)	ANOVA	0.648		0.7	5,34						
C4	Kruskal-Wallis	0.725	2.8		5						
RH	Kruskal-Wallis	0.035 *	12.0		5	Wilcoxon	0.630	1.000	1.000	1.000	1.000
SH	Kruskal-Wallis	0.341	5.7		5						
PF	Kruskal-Wallis	0.618	3.5		5						
AN	Kruskal-Wallis	0.197	7.3		5						
PF_density_m2	Kruskal-Wallis	0.642	3.4		5						
RH_density_m2	Kruskal-Wallis	0.075	10.0		5						
SH_density_m2	Kruskal-Wallis	0.585	3.8		5						
AN_density_m2	Kruskal-Wallis	0.191	7.4		5						

SAGE big sagebrush (% cover), SAGE_height_cm big sagebrush height (cm), SAGE_live_density_m2 big sagebrush density (m^2), SAGE_dead_density 100% dead big sagebrush density (m^2), PG perennial grass (% cover), C3 C_3 perennial bunchgrass (% cover), BG bare ground (% cover), WL woody litter (% cover), HL herbaceous litter (% cover), C3_BU_density_m2 C_3 perennial bunchgrass density (m^2), C4_density_m2

C_4 perennial bunchgrass density (m^2), C4 C_4 perennial bunchgrass (% cover), C4 C_4 perennial bunchgrass (m^2), RH rhizomatous perennial grass (% cover), SH other shrub (% cover), PF perennial forb (% cover), AN annual (% cover), PF_density_m2 perennial forb density (m^2), RH_density_m2 rhizomatous perennial grass (m^2), SH_density_m2 other shrub density (m^2), AN_density_m2 annual density (m^2),

References

Guijarro, J.A. 2016. Climate Tools (Series Homogenization and Derived Products) R-Package Version 3.0. http://www.climatol.eu/.

PRISM Climate Group. 2004. Oregon State University, https://prism.oregonstate.edu.

Walter, H., and H. Leith. 1967. *Klimadiagramm-Weltatlas*. VEB Gustav Fischer Verlag, Jena, Germany.
